# Association between memory impairment and brain metabolite concentrations in North Korean refugees with posttraumatic stress disorder

**DOI:** 10.1371/journal.pone.0188953

**Published:** 2017-12-07

**Authors:** Jung Eun Shin, Chi-Hoon Choi, Jong Min Lee, Jun Soo Kwon, So Hee Lee, Hyun-Chung Kim, Na Young Han, Soo-Hee Choi, So Young Yoo

**Affiliations:** 1 Department of Psychiatry, Seoul National University Hospital, Seoul, Republic of Korea; 2 Department of Radiology, Chungbuk National University Hospital, Cheongju, Republic of Korea; 3 Computational NeuroImage Analysis Laboratory, Department of Biomedical Engineering, Hanyang University, Seoul, Republic of Korea; 4 Department of Psychiatry and Institute of Human Behavioral Medicine in SNU-MRC, Seoul National University College of Medicine, Seoul, Republic of Korea; 5 Department of Psychiatry, National Medical Center, Seoul, Republic of Korea; Max Planck Institute for Psychiatry, GERMANY

## Abstract

Individuals with posttraumatic stress disorder (PTSD) had experiences of enormous psychological stress that can result in neurocognitive and neurochemical changes. To date, the causal relationship between them remains unclear. The present study is to investigate the association between neurocognitive characteristics and neural metabolite concentrations in North Korean refugees with PTSD. A total of 53 North Korean refugees with or without PTSD underwent neurocognitive function tests. For neural metabolite scanning, magnetic resonance spectroscopy of the hippocampus and anterior cingulate cortex (ACC) has been conducted. We assessed between-group differences in neurocognitive test scores and metabolite levels. Additionally, a multiple regression analysis was carried out to evaluate the association between neurocognitive function and metabolite levels in patients with PTSD. Memory function, but not other neurocognitive functions, was significantly lower in the PTSD group compared with the non-PTSD group. Hippocampal N-acetylaspartate (NAA) levels were not different between groups; however, NAA levels were significantly lower in the ACC of the PTSD group than the non-PTSD group (t = 2.424, *p* = 0.019). The multiple regression analysis showed a negative association between hippocampal NAA levels and delayed recall score on the auditory verbal learning test (β = -1.744, *p* = 0.011) in the non-PTSD group, but not in the PTSD group. We identified specific memory impairment and the role of NAA levels in PTSD. Our findings suggest that hippocampal NAA has a protective role in memory impairment and development of PTSD after exposure to traumatic events.

## Introduction

Posttraumatic stress disorder (PTSD) is a psychiatric condition with enormous disability that occurs following exposure to traumatic events such as wars, assault, and natural disasters. PTSD symptoms include re-experiencing of the event, avoidance, and hyperarousal [1]. Without appropriate treatment, PTSD can lead to psychiatric comorbidities such as suicide, depression, anxiety, and personality change [2,3]. It is usually difficult for individuals with PTSD to return to daily life and adapt to jobs or the community [4].

Along with a group of clinical symptoms, memory impairment is a core feature of PTSD [5]. Neuropsychological studies have consistently shown impaired declarative memory [6, 7]and verbal memory [8] in patients with PTSD. However, the impairment of cognitive functions other than memory have returned conflicting results due to different methodologies, diverse target groups, and varying sample sizes [9–13]. Cognitive changes are generally associated with brain dysfunction, and several studies have investigated PTSD on the neural level [14].

Magnetic resonance spectroscopy (MRS) is a noninvasive technique used to study cellular metabolites and the roles they may play in psychiatric disorders [15]. For example, the absence or reduced levels of N-acetylaspartate (NAA), a marker of neuronal/axonal viability, is an indicator of neuronal loss or degradation [16]. Creatine (Cr) is a marker of intracellular metabolism, myo-inositol (Ins) may represent a glial marker, and glutamate-glutamine (Glx) is a marker of neuronal activity in cortical areas [17]. Previous studies have consistently found decreased NAA levels in the hippocampus [18] and anterior cingulate cortex (ACC) [19,20] of patients with PTSD. Low levels of NAA in these brain regions correspond with the etiology and symptomatology of PTSD, given the involvement of the hippocampus in memory and ACC in higher cognitive function. From a neurobiological perspective, the hippocampus responds to stress with increased corticosteroids levels and upregulation of corticosteroid receptors [6,21–23]. The stress response to traumatic events may adversely affect neuronal metabolism, cell survival, and physiological function in the hippocampus [24–26].

In relation to the relationship between neuronal metabolites and cognitive function, a previous rat study has reported a reduced concentration of hippocampal NAA was related to a lack of spatial cognitive learning and memory function in depression [27]. The hippocampal NAA was found to measure cognitive function more sensitively than hippocampal volume. Human study of patients with depression who receive chronic corticosteroid therapy showed significant reductions in hippocampal NAA, auditory verbal memory, and other cognitive functions [28]. However, the authors did not investigate the relationship between variables. Previous studies on sleep apnea associated with intermittent hypoxia and cognitive decrements have found that lower hippocampal NAA and Cr levels were correlated with worse sustained focus attention [29]. Similar associations between neural metabolites and altered cognition may be present in individuals with PTSD.

We investigated the neuropsychological functions, brain metabolite levels, and associations between metabolite levels and neurocognitive function in patients with PTSD. North Korean refugees who had experienced numerous traumatic events before and during the escape from North Korea [30] and settled in South Korea participated in this study. They survived life-threatening conditions such as suppression of human rights, fear of being discovered by security police, and hardships related to living in a third country [31, 32]. We hypothesized that North Korea refugees with PTSD would have neuropsychological impairments, particularly memory dysfunction, and reduced NAA concentrations in the hippocampus and ACC. Additionally, we expected that changes in metabolite levels would have an association with cognitive dysfunctions.

## Materials and methods

### Subjects

The study included 53 North Korean refugees (30 with PTSD and 23 without PTSD) currently living in South Korea from December 2012 to December 2013. Two subjects were excluded from data analysis because they did not take the psychological test, and one did not undergo MRS scanning. The study was approved by the Institutional Review Board of the National Medical Center and all subjects provided written informed consent.

### Demographic and clinical assessments

We obtained data on sociodemographic characteristics, including age, sex, and education from the participants. Additionally, the participants were administered questionnaires concerning their medical history and experiences before entering South Korea, such as physical symptoms, experience of arrest, being captured and returned to North Korea, imprisonment, length of residence in South Korea, and current physical condition and medical treatments (S1 Appendix).

The Clinician-Administered PTSD Scale for DSM-IV (CAPS-DX) was used to evaluate PTSD on three criteria involving (B) re-experience, (C) avoidance, and (D) arousal. The frequency and intensity of each traumatic event were rated on a five-point scale from never/none (0) to always/severe (4). Participants were diagnosed with PTSD when they met three requirements; (1) more than one symptom of re-experience, more than three symptoms in avoidance, and more than two symptoms in arousal criteria, and each symptom was defined when it has at least 1–1 frequency and intensity score combination; (2) more than one month duration; (3) existence of clinically significant distress or functional impairment. The severity score for each criterion was calculated by adding the frequency and intensity scores, and the total score is calculated by adding the severity score for all three criteria [33]. The Minnesota Multiphasic Personality Inventory-PTSD (MMPI-PTSD) was developed to identify PTSD symptoms in combat soldiers and included 45 questions related to PTSD taken from the MMPI questionnaire [34]. Higher scores indicate a higher likelihood of being diagnosed with PTSD.

The Beck Depression Inventory (BDI) was used to measure the severity of depression, including cognitive, emotional, motivational, and physiological symptoms via a self-report questionnaire [35]. Responses to each question were scored on a four-point scale ranging from mild (0) to severe (3). Higher scores indicate more severe depression. The State-Trait Anxiety Inventory (STAI) was administered to assess anxiety. The inventory consists of two sets of 20 questions that measure temporary state and long-lasting anxiety, respectively [36]. The two types of anxiety were scored on a four-point scale ranging from not at all (1) to very much (4), with higher score indicating greater levels of anxiety.

### Neurocognitive function tests

The Korean version of the Wechsler Adult Intelligence Scale (K-WAIS) was used to measure overall cognitive function [37,38]. The instrument consists of 11 subsets, of which 6 are verbal tests and the remaining 5 are performance tests. The total score was converted into the intelligence quotient, a standardized scale with a mean of 100 ± 15.

The Rey—Kim memory test consists of the standardized Auditory Verbal Learning Test (AVLT) [39] and the Complex Figure Test (CFT) [40], which measure verbal and visual memory, respectively. Total scores were converted into the memory quotient, a standardized scale with a mean of 100 ± 15 [41].

The Executive Intelligence Test (EXIT) measures higher cognitive function by testing four cognitive factors: attention, language, visuospatial function and memory [42,43]. The test includes quantitative and qualitative assessments, and the quantitative and qualitative scores are added to obtain the executive intelligence quotient, a standardized scale with a mean ± standard deviation (SD) of 100 ± 15.

### Magnetic resonance imaging (MRI) and MRS acquisition

**Fig 1:**
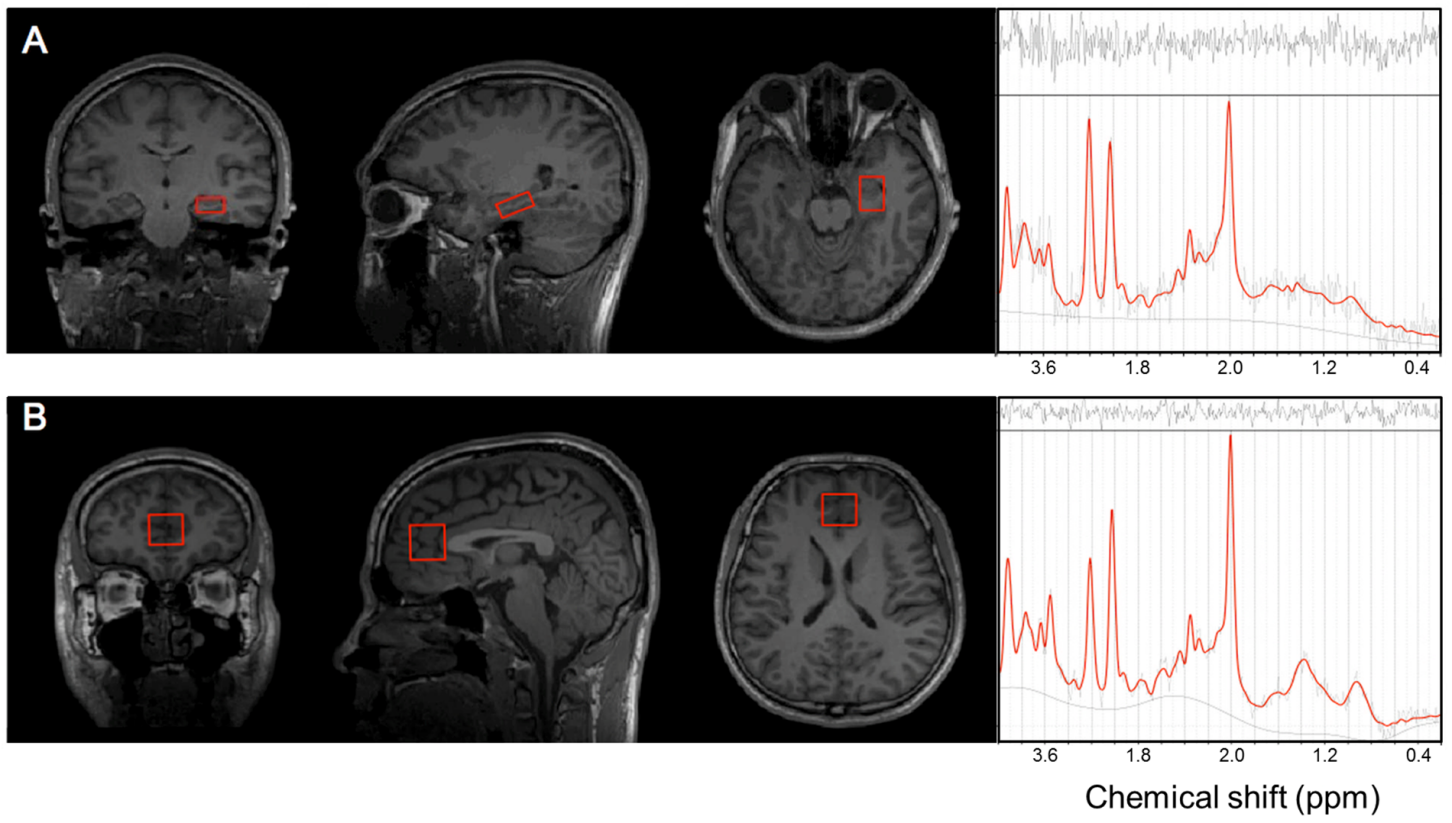
Anatomical location of volumes of interest (VOIs) for magnetic resonance spectroscopy and sample spectra. A: The left hippocampus. B: The anterior cingulate cortex.

All MRI and MRS scannings were performed using a Philips 3.0 Tesla system (Achieva, USA). For ^1^H-MRS volume location, anatomical T1-weighted, 3D whole-head MRI data sets were measured by using a turbo field echo SENSE sequence (repetition time [TR] = 9.8 ms, echo time [TE] = 4.6 ms; sagittal 1 mm thick slices, FOV_APxFH_ 250 × 250 mm^2^). Two volumes of interest (VOIs) were obtained from the hippocampus and ACC (Fig 1). The VOI in the left hippocampus (2 × 1.5 × 1 cm^3^) was placed along the axis of the hippocampus to cover most of the volume. A 2 × 2 × 2 cm^3^ VOI in the ACC was aligned perpendicularly to the tip of the genu of the corpus callosum and centered at the interhemispheric fissure.

A single voxel ^1^H MR spectra comprising 16 water-unsuppressed and 128 water-suppressed averages were acquired using a pointed resolved spin echo spectroscopy pulse sequence (TR = 2,000 ms, TE = 36 ms; scan time per region = 13 min). The raw data from each acquisition consisted of 1,024 points at a bandwidth of 2,000 Hz. The automatic shimming procedure provided by the Philips system was performed for each scan.

Spectroscopic data were analyzed using LCModel software in the range of 4.2–1.0 ppm. LCModel has been used for the identification of low-concentration or overlapping metabolites. To ensure a high-quality spectra, we verified the full-width half-maximum (FWHM) of each two VOIs according group; (1) Hippocampus, PTSD, 0.063 (SD = 0.017; range = 0.031–0.107); non-PTSD, 0.06 (0.015; 0.038–0.092); (2) ACC, PTSD, 0.073 (0.034; 0.038–0.183); non-PTSD, 0.065 (0.027; 0.038–0.138). Cr, Glu, Ins, NAA, Glx, and NAA+N-acetylaspartylglutamate (NAAG) were analyzed and expressed in institutional units as defined in the LCModel manual (www.s-provencher.com/pub/LCModel/manual/manual.pdf). We used the absolute value of metabolites because previous findings suggest that Cr concentration itself may change in subjects [44, 45]. The fitting error of each metabolite was estimated in percent SD. For robustness of final results, only metabolite values with an error < 25% SD were included in the final analysis. The number of subjects who passed this threshold was presented for each metabolites; (1) Hippocampus, Glu (PTSD, n = 25; non-PTSD, n = 20); NAA (PTSD, n = 28; non-PTSD, n = 21); Glx (PTSD, n = 26; non-PTSD, n = 19); (2) ACC, Cr (PTSD, n = 23; non-PTSD, n = 17); Glu (PTSD, n = 29; non-PTSD, n = 23); Ins (PTSD, n = 30; non-PTSD, n = 23); NAA (PTSD, n = 29; non-PTSD, n = 22); NAA+ NAAG (PTSD, n = 30; non-PTSD, n = 23).

### Statistical analysis

Demographic and clinical variables, neuropsychological test scores, and absolute metabolite values were compared between groups using chi-square tests for dichotomous variables and t-tests or Mann-Whitney U tests for continuous variables depending on normality. Multiple linear regression analyses were performed to assess the predictive value of metabolite concentrations for impaired cognitive functions in each of the PTSD and non-PTSD groups, assuming that the two groups had different etiologies in neuronal/psychological changes. The significance level was set at *p* < 0.05. SPSS software (ver. 22.0; SPSS Inc., Chicago, IL, USA) was used to conduct the statistical tests.

## Results

### Demographic and clinical characteristics

The demographic and clinical characteristics of the participants are listed in Table 1 and S1 Table. The frequencies of arrest, being sent back to North Korea, and imprisonment were higher in the PTSD group than in the non-PTSD controls. Participants with PTSD had higher scores on the CAPS-DX and MMPI-PTSD than those without PTSD. The PTSD group had higher frequency of current psychiatric medications than the non-PTSD group. Refugees with PTSD had higher BDI scores with a trend toward significance. We found that there was no difference between groups in level of age (PTSD group, rage = 31–62, median = 47 year; non-PTSD group, range = 24–66, median = 43 year; 95% Confidence Interval of the difference, -2.333, 7.269), gender, education, medical history in North Korea, length of residence of a third country or South Korea, current physical condition and medical treatment, or subjective anxiety as measured by the STAI.

**Table 1:** Demographic and clinical characteristics of participants.

Variables	PTSD (n = 30)		non-PTSD (n = 23)		t/χ^2^	*p* value
	N	%	N	%		
Gender					0.596	0.624
Male	3	10.0	1	4.3		
Female	27	90.0	22	95.7		
Education^a^					1.799	0.180
High school	23	76.7	20	90.9		
College	7	23.3	2	9.1		
Psychiatric symptoms in North Korea (yes)^a^	9	31.0	5	21.7	0.563	0.453
Arrest experience (yes)	21	70.0	5	21.7	12.133	<0.001*
Experience of resending to North Korea (yes)	13	43.4	3	13.0	6.890	0.032*
Prison experience (yes)^a^	16	55.2	5	21.7	5.955	0.015*
Current physical symptoms (yes)	21	70.0	16	69.6	0.001	0.973
Current medical treatment (yes)^c^	18	69.2	15	65.2	0.089	0.765
Current psychiatric medications (yes)	14	46.7	2	8.7	8.907	0.003*
	**Mean**	**SD**	**Mean**	**SD**		
Age (years)	47.0	7.00	44.6	10.39	1.032	0.307
Length of stay in a third country (months)^b^	54.8	51.20	35.6	37.54	0.942	0.346
Length of residence in the South Korea (years)^b^	4.4	2.67	5.1	3.33	0.795	0.427
CAPS-DX	51.7	24.45	13.5	6.33	5.958	<0.001*
MMPI-PTSD	29.5	5.63	20.5	8.32	4.453	<0.001*
BDI	31.0	10.68	25.0	10.74	1.997	0.051
STAI state^a^	51.9	14.71	50.8	13.51	0.279	0.782
STAI trait	53.0	12.96	49.5	13.20	0.944	0.350

Abbreviations: PTSD, post-traumatic stress disorder; SD, standard deviation; CAPS-DX, Clinician-Administered PTSD Scale for DSM-IV; MMPI, Minnesota Multiphasic Personality Inventory; BDI, Beck Depression Inventory; STAI, State-Trait Anxiety Inventory.

* *p* < 0.05;

^a^ one participant missing;

^b^ five participants missing;

^c^ four participants missing.

### Neurocognitive functions and metabolite concentrations

Compared with the non-PTSD group, the refugees with PTSD had lower overall scores on the memory tests, including the total memory quotient score, AVLT-immediate and -delayed recalls, AVLT-delayed recognition, and CFT-immediate recall scores (Table 2). In contrast, there were no significant between-group differences in general cognition (K-WAIS scores) or executive function (EXIT scores).

**Table 2:** Neuropsychological functions of participants.

Measure	PTSD (n = 30)		non-PTSD (n = 23)		t/z	*p* value
	Mean	SD	Mean	SD		
K-WAIS						
Full-scale IQ	91.9	10.49	96.0	13.42	1.239	0.221
Verbal IQ	92.1	11.49	96.1	14.15	1.135	0.262
Performance IQ	92.8	9.56	96.3	12.73	1.204	0.229
Rey-Kim memory test						
MQ	100.1	12.80	109.1	14.17	2.210	0.027*
AVLT-immediate recall	7.5	2.35	8.5	1.86	2.302	0.021*
AVLT-delayed recall	7.1	2.28	8.5	2.78	1.997	0.046*
AVLT-delayed recognition	7.9	2.61	9.6	2.73	2.287	0.022*
CFT-immediate recall	8.3	1.83	10.1	3.07	2.381	0.017*
CFT-delayed recall	8.4	2.03	9.7	3.31	1.740	0.082
Executive Intelligence Test						
Executive IQ	101.8	17.10	106.7	16.64	1.157	0.252
EIQ quality	42.6	5.02	43.6	4.70	0.786	0.432
EIQ quantity	33.2	6.83	34.9	6.64	0.938	0.353

Abbreviations: PTSD, post-traumatic stress disorder; SD, standard deviation; K-WAIS, Korean Wechsler Adult Intelligence Scale; IQ, Intelligence Quotient; MQ, Memory Quotient; AVLT, Auditory Verbal Learning Test; CFT, Complex Figure Test.

* *p* < 0.05.

Table 3 shows group comparison of the absolute values of metabolites. Hippocampal NAA concentration was not significantly different between groups; however, NAA levels were lower in the ACC of participants with PTSD than in the non-PTSD group. The hippocampal and ACC concentrations of the other metabolites did not differ significantly between groups.

**Table 3:** Metabolites in the hippocampus and anterior cingulate cortex of participants.

Variables	PTSD		non-PTSD		t/z	*p* value
	Mean	SD	Mean	SD		
Hippocampus						
Glu	9.791	3.954 (n = 25)	10.506	2.947 (n = 20)	0.672	0.505
NAA	6.007	0.952 (n = 28)	6.297	0.895 (n = 21)	1.083	0.284
Glu+Gln	15.885	6.303 (n = 26)	16.588	5.295 (n = 19)	0.395	0.695
ACC						
Cr	4.179	1.225 (n = 23)	3.865	1.040 (n = 17)	0.854	0.399
Glu	8.919	1.208 (n = 29)	8.823	1.986 (n = 23)	0.202	0.841
Ins	4.811	1.103 (n = 30)	4.746	1.086 (n = 23)	0.214	0.831
NAA	6.365	0.572 (n = 28)	6.736	0.443 (n = 20)	2.424	0.019*
NAA+NAAG	6.799	0.780 (n = 29)	6.851	0.938 (n = 22)	0.305	0.760

Abbreviations: PTSD, post-traumatic stress disorder; SD, standard deviation; Glu, Glutamic acid; NAA, N-Acetylaspartate; Gln, Glutamine; ACC, Anterior Cingulate Cortex; Cr, Creatine; Ins, Myo-inositol; NAAG, N-acetylaspartylglutamate.

* *p* < 0.05.

### Association between memory function and metabolite concentrations

We performed a multivariate linear regression analysis to evaluate the association between impaired memory function and metabolite concentrations using significant items from the Rey-Kim test as dependent variables: AVLT-immediate, AVLT-delayed recall, AVLT-delayed recognition, and CFT-immediate recall scores and using metabolites in the hippocampus and ACC as independent variables. The model consisted of the metabolites that survived a stepwise regression and was adjusted for age and BDI scores, which is well known for its impact on cognitive functions. The coefficients of determination (R^2^) of the multivariate regression analysis models, with memory test scores as the dependent variables, ranged from 0.06 to 0.41 (Table 4). The analysis revealed a negative association between NAA concentration in the hippocampus and AVLT-delayed recall in the refugees without PTSD (S1 Fig), whereas NAA concentrations in the hippocampus and ACC were not significantly associated with AVLT-delayed recognition, AVLT-immediate recall or CFT-immediate recall.

**Table 4:** Multivariate linear regression models of the associations between metabolite levels and memory function.

Variables	AVLT-immediate recall			AVLT-delayed recall			AVLT-delayed recognition			CFT-immediate recall		
	Coeff	SD	*p* value	Coeff	SD	*p* value	Coeff	SD	*p* value	Coeff	SD	*p* value
PTSD												
NAA, hippocampus	-	-	-	-0.083	0.464	0.859	-	-	-	0.164	0.378	0.668
Cr, ACC	-0.011	0.466	0.959	-	-	-	0.231	0.504	0.652	-	-	-
Age	-0.129	0.095	0.556	-0.073	0.061	0.243	-0.057	0.103	0.587	0.016	0.050	0.756
BDI	-0.349	0.054	0.130	-0.049	0.040	0.230	-0.053	0.058	0.368	-0.041	0.032	0.216
Partial R^2^	0.017			-0.005			-0.058			-0.043		
*p* value (model)	0.364			0.429			0.626			0.601		
non-PTSD												
NAA, hippocampus	-	-	-	**-1.744**	**0.608**	**0.011**	-	-	-	-1.420	0.897	0.132
Cr, ACC	-0.564	0.409	0.191	-	-	-	-1.114	0.59	0.082	-	-	-
Age	-0.076	0.041	0.086	-0.04	0.051	0.439	-0.124	0.059	0.056	0.003	0.075	0.969
BDI	-0.027	0.037	0.472	-0.065	0.043	0.153	0.024	0.053	0.664	-0.069	0.064	0.297
Partial R^2^	0.267			0.410			0.362			0.057		
*p* value (model)	0.073			0.007			0.032			0.275		

Abbreviations: AVLT, Auditory Verbal Learning Test; CFT, Complex Figure Test; Coeff, coefficient; SD, standard deviation; NAA, N-Acetylaspartate; ACC, Anterior Cingulate Cortex; Cr, Creatine; BDI, Beck Depression Inventory

## Discussion

The results demonstrate memory impairment among other neurocognitive functions in refugees with PTSD. Additionally, there was a lack of associations between brain metabolites of hippocampal NAA and memory function in refugees with PTSD compared to refugees without PTSD. We found that the neuropsychological tests revealed that refugees with PTSD performed worse on memory tasks, but did not differ significantly from those without PTSD on the general cognition or higher cognitive functions. Previous evidence suggests that impaired memory may be a higher priority than other executive functions in individuals with PTSD [46]. The study of memory in individuals with PTSD found that those with low memory test scores performed poorly on cognitive function tests, whereas cognitive function in those with higher memory scores was similar to that of controls. Impaired memory function may manifest earlier than other cognitive deficits in refugees with PTSD and that may be associated with hippocampal dysfunction caused by traumatic stress.

In relation to memory function, we found that both immediate and delayed verbal memory scores were lower in the refugees with PTSD, whereas only immediate recall in visual memory was impaired in this group. A previous meta-analysis found that impaired verbal memory was the most consistent cognitive impairment related to PTSD [8]. Furthermore, another meta-analysis found greater impairment in verbal than in visual memory in individuals with PTSD [47]. Our finding of lower verbal memory scores in refugees with PTSD is consistent with those of previous studies. The relationship between PTSD and visual memory is controversial: one previous study found differences in visual memory between the general public and combat veterans [48], whereas, others found no difference between participants with and those without PTSD [49, 50]. Thus, although previous findings suggest that traumatic events have a greater effect on verbal than visual memory, further research on visual memory is needed to confirm earlier findings.

Previous MRS studies of neural metabolites have consistently found reduced NAA levels in the hippocampus and ACC of patients with PTSD [20, 51]. Our finding of decreased NAA levels in the ACC, but not hippocampus, was in partial agreement with previous studies. Because a considerable number of non-PTSD refugees in the comparison group also had subclinical PTSD symptoms, the two groups would show comparable hippocampal NAA levels. However, we found between-group differences in the association between NAA level and memory function. We found that low scores in the delayed recall test were associated with higher levels of hippocampal NAA in refugees without PTSD, whereas there was no association between hippocampal NAA level and verbal delayed recall memory in refugees with PTSD. This suggests that preserved memory function of non-PTSD refugees, despite of traumatic events, can be attributed to an intact role of hippocampal NAA. Lack of this relationship between hippocampal NAA level and memory function in refugees with PTSD would be one of underpinnings in development of PTSD.

A previous study investigating the preventative effect of NAA on PTSD suggested that pre-trauma hippocampal NAA levels were a predictor of susceptibility to PTSD-related symptoms in mice [52]. Animals with high pre-traumatic NAA levels decreased their fear reaction to control levels during re-exposure to traumatic cues. The associations between pre-traumatic levels of brain metabolite and PTSD have not been investigated in human studies. In patients with PTSD, the posttraumatic levels of the metabolite have been measured on the assumption that the traumatic event had altered the NAA level. Meanwhile, it has been reported that hippocampal NAA concentration is positively correlated with cortisol level in subjects without hypercortisolemia, suggesting that cortisol has a trophic effect on hippocampal neurons within normal range. [53]. Many studies have shown the low cortisol level in patients with PTSD [54, 55] and adult offspring [56]. Considering that cortisol plays a key role in the learning and memory in the central nervous system [57], it can be postulated that decreased in cortisol level after traumatic event may lower the hippocampal NAA concentration and impaired memory function, associated with development of PTSD. The reason of not all people who are exposed to traumatic events have PTSD has not yet been resolved. There may be individual vulnerabilities such as genetic variability, sex differences, and developmental trauma exposures [58]. In light of previous findings, it can be said that higher post-trauma NAA concentrations in the hippocampus are protective for impaired memory function and prevent progression to PTSD.

The significance of the reduced NAA level in the ACC in the PTSD group is supported by the fact that this brain region plays a role in selective attention and extinction of the fear response [59, 60]. Moreover, the ACC modulates neuronal activity in the hippocampus and amygdala via afferent projections [61]. ACC dysfunction, thus, may cause PTSD symptoms, such as persistent hyper-reactivity to traumatic events. Functionally, neuronal activity of the ACC in patients with PTSD has been shown to be decreased compared with that of controls [62]. Our finding of lower NAA levels in the ACC suggests that its modulatory effect of neuronal activity on the hippocampus and amygdala is impaired in refugees with PTSD.

North Korean refugees with PTSD were more likely to have experiences of being arrested, imprisoned, or captured and sent back to North Korea than refugees without PTSD. During their escape, many refugees were under surveillance by North Korean guards, experiencing fear of detection and food shortages. [31]. Many nearly died and experienced excessive life-threating stress and trauma until they were able to escape again. These may contribute to the development of PTSD in neural and cognitive level in North Korean refugees.

Our study had several limitations. First, our dataset have limited properties. It is composed of relatively small sample size and is skewed toward females, focusing on the cross-sectional perspective. In addition, this clinical data is not feasible to address all of the various human factors that affect possible cognitive functions and brain metabolites, precluding an inference of the cause-effect relationships. However, given that 71% of North Korean refugees currently consist of females [63] and PTSD is more prevalent among females than among males across the lifespan, gender ratio of this study seems to be ecologically plausible in both epidemiological and clinical aspects. Second, a considerable number of participants in the non-PTSD group had some PTSD symptoms, and only nine participants had no PTSD symptoms in the present study. To extend the present findings, further studies are needed on larger populations and subgroup without psychiatric symptoms of PTSD. Third, the control group of North Koreans who are not refugees and are not exposed to stress might strengthen the present findings. Because of the political circumstances, this problem remains as a limitation that cannot be solved at present. Fourth, we did not collect data on alcohol consumption. Individuals with PTSD are more likely to abuse alcohol than non-PTSD patients, and because alcohol consumption may have a permanent effect on the hippocampus and other brain regions [64], future studies taking alcohol assumption into consideration are warranted.

In conclusion, we found memory impairment among other neurocognitive functions and lack of specific relationship between hippocampal NAA and memory function in refugees with PTSD. Refugees without PTSD had an inverse association between hippocampal NAA levels and memory function, suggesting key role of NAA levels in preventing memory impairment and development of PTSD in individuals with experience of traumatic events. Our findings confirm a distinctive deficit in memory function and highlight the important role of NAA in the hippocampus and ACC in PTSD.

## Supporting information

S1 Appendix Survey questionnaires. (DOCX)

S1 Table Participant characteristics. (DOCX)

S1 Fig Partial correlation between AVLT-delayed recall score and hippocampal NAA concentration using age and BDI scores as covariates. Abbreviations: AVLT, Auditory Verbal Learning Test; NAA, N-Acetylaspartate; BDI, Beck Depression Inventory.
(TIFF)
