# Supplementary material for: Association between memory impairment and brain metabolite concentrations in North Korean refugees with posttraumatic stress disorder
Source: PLoS One. 2017 Dec 7;12(12):e0188953. doi: 10.1371/journal.pone.0188953 (PMC5720673; doi:10.1371/journal.pone.0188953)
Supplement: S1 Appendix — (DOCX) [file pone.0188953.s001.docx]

**S1 Appendix. Survey questionnaires.**

Initials : __________ Age : _____ Gender : M / F Highest Degree Received:

Date : __________

**The following questions are about living in North Korea.**

1. What was your educational background in North Korea?

① None ② Elementary school dropout ③ Elementary school graduation

④ Middle/high school dropout ⑤ Middle/high school graduation

⑥ More than Bachelor's degree

2. Describe your economic status in North Korea.

① Good ② Average ③ Bad

3. What was your occupation in North Korea? ( )

4. Did you have psychiatric symptoms in North Korea?

① Yes ② No

If yes, what did you do to alleviate your symptoms?

1. Chinese medicine ② Medical care ③ Psychiatric care

④ Convalescence (rest) ⑤ Other (write it down) ___________

5. Have you ever received any psychiatric or psychological treatment in North Korea?

① Yes ② No

If yes, please provide how many times of psychiatric treatment you received and what was the diagnosis of mental disorders you had in North Korea.

- Number of psychiatric treatment ( )

- Diagnosis of mental disorders ( )

**The following questions are about your experience during the escape process.**

6. When was the first time you left North Korea? Please specify year. ______

7. When was the last time you left North Korea? Please specify year. ______

8. Did you live other countries else before coming to South Korea? ① Yes ② No

If yes, which country did you stay?

1. China ② Thailand ③ Laos ④ Myanmar ⑤ Mongol ⑥ Russia ⑦ Other

If yes, how long have you stayed in the countries?

Please specify the duration : years months

9. Have you ever been caught by a Chinese public security during the escape process?

① Yes ② No

10. Have you ever been sent back to North Korea during the escape process?

① Yes ② No

11. Have you been confined in a correctional facility or a prison during the escape process? ① Yes ② No

**The following questions are about living in South Korea.**

12. When did you come to South Korea? (By year of entry) year

13. Please check if you have any families or friends in South Korea.

① None ② Spouse ③ Parents ④ Children ⑤ Grandparents
⑥ Grandchildren ⑦ Relatives ⑧ Friends

14. Please check if you still have any family members in North Korea.

① None ② Spouse ③ Parents ④ Children ⑤ Grandparents
⑥ Grandchildren ⑦ Relatives ⑧ Friends

15. Do you currently have a job? ① Yes ② No

If yes, please write it down.

16. Do you currently have physical symptoms? ① Yes _________ ② No

If yes, please write it down. __________________

17. Are you currently undergoing any medical treatment? ① Yes ② No

If yes, please write it down. __________________

18. Is it difficult to understand the South Korean language?

① Not at all ② Little difficult ③ Difficult ④ Very difficult

19. Is it difficult to get along with South Koreans?

① Not at all ② Little difficult ③ Difficult ④ Very difficult

Please specify if you have any difficulty. ___________________________

20. Have you ever had the feeling or experience of being ignored by South Koreans?

① Never ② Occasionally ③ Often ④ Mostly

21. Please write if you have any other difficulties in living in South Korea.

________________________________________________________________________

이름 : __________ 연령 : _____세 성별 : 남 / 녀 최종 학력:

작성일 : 년 월 일

**다음은 북한 생활에 대한 질문입니다.**

1. 북한에서의 학력은?

① 학교를 안다님 ② 인민학교(소학교) 중퇴 ③ 인민학교(소학교) 졸업

④ 고등중학교 중퇴 ⑤ 고등중학교 졸업 ⑥ 대학 이상

2. 북한에서의 경제 상태는?

① 상 ② 중 ③ 하

3. 북한에서의 직업은? ( )

4. 정신건강의학과(정신과)에 오게 된 증상이 과거 북한에서 있었습니까 ?

① 있었다 ② 없었다

만약 ‘있었다’고 대답하셨다면, 증상을 완화하기 위하여 어떠한 행동을 하셨습니까?

① 전통편 복용 ② 내과 진료 ③ 정신과 진료

④ 요양(휴양) ⑤ 기타(적어주세요) ___________

5. 북한에서 본인의 정신건강의학과(정신과)의 치료 경험이 있습니까?

① 있다 ② 없다

만약 ‘예’라고 대답하셨다면, 북한에서 본인의 정신건강의학과(정신과)의 치료 경험이 있다면 몇번이고 병명은 무엇입니까? ( ) 회 / 병명 ( )

**다음은 탈북 과정 중 경험에 대한 질문입니다.**

6. 북한에서 언제 최초로 나왔습니까? 년

7. 북한에서 언제 마지막으로 나왔습니까? 년

8. 남한에 입국하기 전에 주변국에 머물렀습니까? ① 예 ② 아니오

만약 ‘예’라고 대답하셨다면, 어느 나라에 머물렀습니까?

① 중국 ② 태국 ③ 라오스 ④ 미얀마 ⑤ 몽골 ⑥ 러시아 ⑦ 기타

만약 ‘예’라고 대답하셨다면, 얼마동안 머물렀습니까? 년 개월

9. 탈북 과정에서 중국 공안에 잡혔던 적이 있습니까? ① 예 ② 아니오

10. 탈북 과정에서 북송되었던 적이 있습니까? ① 예 ② 아니오

11. 탈북 과정에서 교화소 혹은 구치소에 갇힌 적이 있습니까? ① 예 ② 아니오

**다음은 현재 남한에서의 생활에 관한 질문입니다.**

12. 남한에 언제(입국기준) 들어왔습니까? 년

13. 남한에 있는 가족이나 친구가 있으면 모두 표시하여 주십시오.

① 전혀없다 ② 배우자 ③ 부모 ④ 자녀 ⑤ 조부모

⑥ 손자 ⑦ 친척 ⑧ 친구

14. 북한에 남아 있는 가족이 있으면 모두 표시하여 주십시오.

① 전혀없다 ② 배우자 ③ 부모 ④ 자녀 ⑤ 조부모

⑥ 손자 ⑦ 친척

15. 현재 직장이 있습니까? ①예 ②아니오

있다면 적어주십시오. __________________

16. 현재 신체적 증상이 있습니까? ①예 ②아니오

있다면 적어주십시오. __________________

17. 현재 치료받고 있는 질환이 있습니까? ①예 ②아니오

있다면 적어주십시오. __________________

18. 남한 언어를 이해하는데 어려움이 있습니까?

① 전혀 없다 ② 조금 어렵다 ③ 어렵다 ④ 매우 어렵다

19. 남한 사람들과 어울리는데 어려움이 있습니까?

① 전혀 없다 ② 조금 어렵다 ③ 어렵다 ④ 매우 어렵다

어려움이 있다면 구체적으로 적어주십시오. __________________

20. 남한 사람들에게 무시를 당하는 것 같은 느낌 혹은 경험을 가진 적이 있습니까?

① 전혀 없다 ② 조금 있다 ③ 종종 있다 ④ 많이 있다

21. 이 외에 남한에 살면서 힘들었던 점이 있다면 적어 주십시오.
