# Supplementary material for: Association between memory impairment and brain metabolite concentrations in North Korean refugees with posttraumatic stress disorder
Source: PLoS One. 2017 Dec 7;12(12):e0188953. doi: 10.1371/journal.pone.0188953 (PMC5720673; doi:10.1371/journal.pone.0188953)
Supplement: S1 Table — (DOCX) [file pone.0188953.s002.docx]

**S1 Table. Participant characteristics.**

| ID | Age | PTSD  diagnosis | Sex | Education_ NK | Symp_NK | Arrest_ exp | Resending  _NK | Prison_ exp | Current  _phys | Current  _med | Current  _psych | Stay  _3rd, months | Stay_ SK, years | CAPS  -DX | MMPI-PTSD | BDI | STAI state | STAI trait |
| --- | --- | --- | --- | --- | --- | --- | --- | --- | --- | --- | --- | --- | --- | --- | --- | --- | --- | --- |
| PTSD01 | 52 | PTSD | F | High school | Y | N | N | N | Y | Y | Y | 1 | 8 | 25 | 31 | 22 | 59 | 55 |
| PTSD02 | 50 | PTSD | F | High school | N | Y | Y | Y | Y | Y | N | 128 | 5 | 24 | 25 | 15 | 52 | 45 |
| PTSD03 | 53 | PTSD | F | High school | N | N | N | N | Y | Y | Y | 24 | 10 | 24 | 31 | 42 | 71 | 72 |
| PTSD04 | 37 | PTSD | F | High school | N | Y | Y | Y | Y | Y | N | 86 | 3 | 92 | 24 | 21 | 22 | 38 |
| PTSD05 | 53 | PTSD | F | College | N | Y | N | N | Y | N | N | - | - | 72 | 22 | 15 | 34 | 34 |
| PTSD06 | 42 | PTSD | F | High school | N | Y | Y | Y | N | Y | N | 81 | 9 | 64 | 35 | 40 | 63 | 73 |
| PTSD07 | 42 | PTSD | F | College | N | Y | N | N | N | N | Y | - | - | 83 | 18 | 27 | 57 | 62 |
| PTSD08 | 31 | PTSD | F | High school | N | Y | N | Y | N | Y | N | 123 | 3 | 41 | 29 | 32 | 52 | 53 |
| PTSD09 | 56 | PTSD | F | College | Y | Y | Y | Y | Y | N | N | 54 | 1 | 84 | 27 | 33 | 52 | 47 |
| PTSD10 | 58 | PTSD | F | College | Y | N | N | N | Y | Y | Y | 14 | 6 | 34 | 35 | 49 | 55 | 67 |
| PTSD11 | 43 | PTSD | F | College | N | N | - | - | N | - | N | 48 | 6 | 32 | 28 | 25 | 48 | 50 |
| PTSD12 | 62 | PTSD | M | High school | Y | Y | Y | Y | Y | N | Y | 120 | 4 | 16 | 16 | 17 | 40 | 42 |
| PTSD13 | 55 | PTSD | F | High school | N | Y | Y | Y | Y | - | Y | 66 | 10 | 34 | 31 | 31 | 56 | 53 |
| PTSD14 | 46 | PTSD | F | High school | N | Y | N | Y | Y | Y | N | 60 | 7 | 31 | 28 | 12 | 56 | 53 |
| PTSD15 | 50 | PTSD | F | High school | Y | Y | Y | Y | Y | Y | N | 132 | 3 | 43 | 23 | 24 | 52 | 52 |
| PTSD16 | 44 | PTSD | F | High school | Y | N | N | N | Y | Y | Y | 4 | 4 | 45 | 34 | 48 | 72 | 59 |
| PTSD17 | 51 | PTSD | F | High school | Y | N | N | N | Y | Y | Y | 2 | 2 | 41 | 38 | 36 | 54 | 51 |
| PTSD18 | 48 | PTSD | F | College | N | N | N | N | Y | Y | Y | 3 | 2 | 42 | 33 | 36 | 74 | 75 |
| PTSD19 | 44 | PTSD | F | High school | Y | Y | Y | Y | N | Y | Y | 78 | 5 | 27 | 26 | 30 | 8 | 24 |
| PTSD20 | 47 | PTSD | F | High school | N | Y | N | N | Y | - | N | 144 | 2 | 72 | 30 | 26 | 55 | 64 |
| PTSD21 | 46 | PTSD | M | High school | - | N | N | N | Y | Y | N | 0 | 3 | 79 | 32 | 41 | 73 | 71 |
| PTSD22 | 46 | PTSD | F | High school | N | Y | N | N | N | N | N | - | - | 27 | 31 | 44 | 66 | 66 |
| PTSD23 | 38 | PTSD | F | High school | N | Y | N | N | N | N | N | - | - | 60 | 24 | 29 | 42 | 42 |
| PTSD24 | 54 | PTSD | F | High school | N | N | N | N | Y | Y | Y | 14 | 1 | 92 | 33 | 30 | 44 | 51 |
| PTSD25 | 46 | PTSD | F | High school | N | Y | Y | Y | Y | N | N | 10 | 2 | 36 | 35 | 42 | - | - |
| PTSD26 | 41 | PTSD | F | High school | N | Y | Y | Y | Y | - | Y | 5 | 6 | 54 | 40 | 50 | 66 | 66 |
| PTSD27 | 34 | PTSD | F | High school | Y | Y | N | Y | Y | Y | Y | 2 | 2 | 53 | 27 | 20 | 42 | 42 |
| PTSD28 | 48 | PTSD | F | College | N | Y | Y | Y | Y | Y | N | 84 | 4 | 84 | 37 | 21 | 43 | 34 |
| PTSD29 | 50 | PTSD | M | High school | N | Y | Y | Y | N | Y | Y | 6 | 2 | 99 | 31 | 40 | 54 | 51 |
| PTSD30 | 44 | PTSD | F | High school | N | Y | Y | Y | N | N | N | 134 | 4 | 41 | 31 | 31 | 44 | 44 |
| Non-PTSD 01 | 64 | None | F | High school | N | Y | N | N | Y | Y | N | 48 | 12 | 16 | 19 | 19 | 47 | 48 |
| Non-PTSD 02 | 45 | None | F | High school | N | Y | N | N | Y | N | N | 33 | 13 | 9 | 18 | 27 | 37 | 37 |
| Non-PTSD 03 | 49 | Subsynd-romal | F | High school | N | N | N | Y | Y | Y | Y | 2 | 4 | 16 | 28 | 44 | 58 | 68 |
| Non-PTSD 04 | 56 | None | F | High school | Y | N | N | N | N | Y | N | - | - | 15 | 15 | 27 | 56 | 46 |
| Non-PTSD 05 | 37 | None | F | High school | N | N | N | N | Y | Y | N | 30 | 4 | 13 | 15 | 31 | 44 | 48 |
| Non-PTSD 06 | 36 | None | F | - | N | N | N | N | N | N | N | 26 | 2 | 6 | 12 | 24 | 42 | 41 |
| Non-PTSD 07 | 37 | Partial | F | High school | N | N | N | N | Y | N | N | 3 | 4 | 21 | 26 | 15 | 65 | 55 |
| Non-PTSD 08 | 53 | Subsynd-romal | F | High school | Y | N | N | Y | Y | Y | N | 5 | 6 | 18 | 33 | 30 | 65 | 67 |
| Non-PTSD 09 | 54 | Subsynd-romal | F | High school | N | N | N | N | Y | Y | N | 18 | 9 | 21 | 8 | 39 | 70 | 46 |
| Non-PTSD 10 | 30 | Subsynd-romal | F | High school | N | N | Y | N | N | N | N | 96 | 4 | 10 | 11 | 32 | 71 | 69 |
| Non-PTSD 11 | 66 | Partial | F | High school | Y | Y | N | N | Y | Y | Y | 18 | 4 | 17 | 32 | 30 | 64 | 60 |
| Non-PTSD 12 | 39 | None | F | High school | N | Y | Y | Y | Y | Y | N | 110 | 5 | 1 | 13 | 5 | 32 | 30 |
| Non-PTSD 13 | 46 | Subsynd-romal | F | High school | Y | N | N | N | Y | Y | N | 10 | 1 | 16 | 21 | 23 | 59 | 55 |
| Non-PTSD 14 | 43 | Subsynd-romal | F | High school | N | N | N | Y | N | Y | N | 60 | 3 | 15 | 34 | 46 | 70 | 72 |
| Non-PTSD 15 | 32 | Subsynd-romal | F | High school | Y | N | N | N | Y | Y | N | 2 | 3 | 22 | 33 | 26 | 55 | 59 |
| Non-PTSD 16 | 55 | None | F | College | N | N | N | N | Y | N | N | 8 | 6 | 9 | 11 | 12 | 42 | 41 |
| Non-PTSD 17 | 42 | Subsynd-romal | F | High school | N | N | N | N | Y | Y | N | 10 | 2 | 12 | 23 | 28 | 43 | 45 |
| Non-PTSD 18 | 43 | Subsynd-romal | F | High school | N | N | N | N | Y | Y | N | 42 | 5 | 18 | 27 | 28 | 41 | 36 |
| Non-PTSD 19 | 45 | None | F | High school | N | N | N | N | Y | Y | N | 126 | 6 | 2 | 25 | 16 | 52 | 54 |
| Non-PTSD 20 | 37 | None | F | College | N | Y | Y | Y | N | N | N | 3 | 11 | 4 | 8 | 4 | 22 | 24 |
| Non-PTSD 21 | 42 | Subsynd-romal | F | High school | N | N | N | N | N | N | N | 49 | 4 | 63 | 25 | 30 | 57 | 62 |
| Non-PTSD 22 | 24 | Partial | F | High school | N | N | N | N | N | N | N | 81 | 1 | 23 | 15 | 26 | 43 | 42 |
| Non-PTSD 23 | 50 | Subsynd-romal | M | High school | N | N | N | N | Y | Y | N | 3 | 4 | 13 | 20 | 14 | 34 | 34 |

Abbreviations: PTSD, Posttraumatic stress disorder; NK, North Korea; Symp_NK, Symptoms in North Korea; Arrest_exp, Arrest experience; Resending_NK, Experience of resending to North Korea; Prison_exp, Prison experience; Current_phys, Current physical symptoms; Current_med, Current medical treatment; Current_psych, Current psychiatric medications; Stay_3rd, Length of Stay in a third country; Stay_SK, Length of residence in the South Korea; CAPS-DX, Clinician-Administered PTSD Scale for DSM-IV; MMPI, Minnesota Multiphasic Personality Inventory, BDI, Beck Depression Inventory; STAI, State-Trait Anxiety Inventory.
