# Supplementary figures and images for: Association between memory impairment and brain metabolite concentrations in North Korean refugees with posttraumatic stress disorder
Source: PLoS One. 2017 Dec 7;12(12):e0188953. doi: 10.1371/journal.pone.0188953 (PMC5720673; doi:10.1371/journal.pone.0188953)

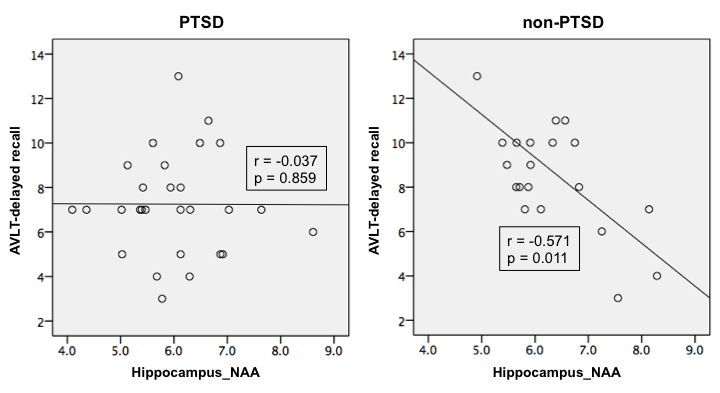

Supplement: S1 Fig — Abbreviations: AVLT, Auditory Verbal Learning Test; NAA, N-Acetylaspartate; BDI, Beck Depression Inventory. (TIFF) [file pone.0188953.s003.tiff]
